# A methoxy derivative of resveratrol analogue selectively induced activation of the mitochondrial apoptotic pathway in transformed fibroblasts

**DOI:** 10.1038/sj.bjc.6602300

**Published:** 2005-01-25

**Authors:** A Gosslau, M Chen, Ci-T Ho, K Y Chen

**Affiliations:** 1 Department of Chemistry and Chemical Biology, Center for Advanced Food Technology, Rutgers, The State University of New Jersey, Piscataway, NJ 08854-8087, USA; 2 New Jersey Cancer Institute, New Brunswick, NJ 08901, USA

**Keywords:** resveratrol, chemoprevention, apoptosis, mitochondria

## Abstract

Resveratrol (R-3), a trihydroxy trans-stilbene from grape, inhibits multistage carcinogenesis in animal models. A resveratrol derivative 3,4,5,4′-tetrahydroxystilbene (R-4) exhibits potent growth inhibitory effect against transformed human cells. Here we report that 3,4,5,4′-tetramethoxystilbene (MR-4), converted from R-4, was more potent against cancer cell lines (WI38VA, IMR-90SV, HeLa, LNCaP, HT-29, and HepG2), but had almost no inhibitory effect on the growth of normal cells (WI38, IMR-90, BJ-T) at the concentrations tested. The IC_50_ value of MR-4 on the growth inhibition of transformed WI38VA human cells was 0.5 *μ*M, as compared to the value of greater than 50 *μ*M for the normal WI38 cells. Resveratrol, however, did not exhibit such clear differential effect and the IC_50_ value of R-3 for WI38VA cells was about 50 *μ*M. The growth inhibitory effect of MR-4 correlated with the induction of apoptosis in the transformed cells. When normal WI38 cells and transformed WI38VA cells were compared, MR-4 induced increases of the Bax/Bcl-2 mRNA ratio, p53 and Bax protein level, activation of caspases, and DNA fragmentation in transformed, but not in normal cells. Further analysis revealed that MR-4 caused a rapid appearance of perinuclear aggregation of mitochondria in WI38VA but not in WI38 cells, suggesting that the mitochondria could serve as an early target of MR-4. R-3 also induced apoptosis and mitochondrial clustering but only at a much higher concentration, close to 500 *μ*M. Taken together, the specific activation of the mitochondria-mediated apoptotic pathway could be a major reason for the striking differential growth inhibitory effect of MR-4.

Resveratrol (3,5,4′-trihydroxy-*trans*-stilbene), a phytoalexin present in grapes, peanuts, and pines, has antioxidant and anti-inflammatory activities (Jeandet *et al*, 1991), and is the active ingredient in Leguminosae that inhibits cellular events associated with tumour initiation, promotion, and progression in mouse skin cancer model (Jang *et al*, 1997). *In vitro*, resveratrol inhibits the growth of various human tumour cells, including oral squamous carcinoma (Elattar and Virji, 1999), promyelocytic leukemia (Surh *et al*, 1999), human breast cancer cells (Lu and Serrero, 1999), prostate cancer cells (Hsieh and Wu, 1999; Narayanan *et al*, 2003), oesophageal carcinoma cells (Zhou *et al*, 2003), pancreatic cancer cells (Ding and Adrian, 2002), and monocytic leukaemia cell (Tsan *et al*, 2000). The potential of resveratrol as a cancer chemoprevention reagent has been extensively reviewed (Gusman *et al*, 2001; Bhat and Pezzuto, 2002; Savouret and Quesne, 2002; Aziz *et al*, 2003). The possible role of resveratrol, a phytoestrogen, in cardiovascular protection has also been discussed recently (Hung *et al*, 2001; Wu *et al*, 2001). Nevertheless, there are two concerns with regard to the use of resveratrol as chemoprevention agent. First, most of the reported studies did not use paired normal and cancer cells to compare the effects of resveratrol. For effective chemoprevention, it is desirable that the candidate compound demonstrates clear differential growth inhibitory effect against cancer cells. Second, the IC_50_ of growth inhibition for resveratrol, which varied between 40 and 200 *μ*M as reported in the literature for various cell types, is relatively high. To address these concerns, we have used a matched pair of normal and transformed human fibroblasts to compare the antiproliferative activity of resveratrol and other analogues derived from resveratrol (Lu *et al*, 2001). We have observed a selective induction of apoptosis in cancer cells by R-4 (3,4,5,4′-tetrahydroxystilbene) (Lu *et al*, 2001).

Apoptosis can be considered as a proactive self-defense mechanism of a living organism to weed out dysfunctional cells such as the precursors of metastatic cancer cells without creating secondary oxidative stress due to inflammation (Davies, 2000). Aberration of apoptotic pathways leads to cancer and other diseases (Igney and Krammer, 2002; Reed, 2003). Apoptosis is defined by a set of energy-dependent changes including chromatin condensation, DNA fragmentation, membrane blebbing, and cell shrinkage. These events lead ultimately to cell death (Green and Reed, 1998; Hengartner, 2000; Reed, 2002; vanGurp *et al*, 2003). There exist two major pathways that initiate apoptosis: the extrinsic (receptor-mediated) and intrinsic (mitochondria-mediated) pathway, which can crosstalk to each other (Green and Reed, 1998; Hengartner, 2000; Reed, 2002; vanGurp *et al*, 2003; Gosslau and Chen, 2004). The extrinsic pathway is triggered by the binding of ligands (e.g. TNF) to their cell death receptors (TNF-receptor) located on the plasma membrane (Strater and Moller, 2000; Peter and Krammer, 2003). Activation is triggered by receptor trimerisation and recruitment of associated proteins, which result in activation of procaspase-8 and caspase-3 activation (Strater and Moller, 2000; Peter and Krammer, 2003; Gosslau and Chen, 2004).

The crucial step in the mitochondria-mediated apoptosis is the change of mitochondrial membrane permeability, and collapse of membrane potential (Green and Reed, 1998; Hengartner, 2000; Reed, 2002; vanGurp *et al*, 2003; Gosslau and Chen, 2004). The change can be induced by (i) an opening of the permeability transition pore (PTP); (ii) an increase of the Bax/Bcl-2 ratio; or (iii) reactive oxygen species (ROS)-induced damage of mitochondrial membrane (Green and Reed, 1998; Hengartner, 2000; Reed, 2002; vanGurp *et al*, 2003; Gosslau and Chen, 2004). The change in membrane permeability causes mitochondrial swelling, rupture of the outer membrane, and release of proapoptotic factors such as cytochrome *c*, pro-caspases, Apaf-1, and AIF, from the intermembranous space, which leads to the formation of a supramolecular apoptosome complex that turns on the caspase cascade (Green and Reed, 1998; vanGurp *et al*, 2003). Increase of the Bax/Bcl-2 ratio is proapoptotic by inducing permeabilisation of mitochondria. Caspase-3 serves as the ‘central executioner of apoptosis’ since it is activated by the extrinsic as well as intrinsic pathway (Susin *et al*, 1997). Caspase-3 activates other caspases, cleaves cytoskeletal proteins (e.g. fodrin and gelsolin), or activates the caspase-activated DNase (CAD), which is involved in fragmentation of nuclear DNA. The characteristic cleavage of DNA into nucleosomal fragments has been regarded as a hallmark of apoptosis (Compton, 1992).

Recently, we reported that among eight resveratrol derivatives, R-4 (3,4,5,4′-tetrahydroxy-*trans*-stilbene) inhibited the growth of transformed cells, but had little effect on the growth of the normal cells (Lu *et al*, 2001). We now report that MR-4 (3,4,5,4′-tetramethoxy-*trans*-stilbene), the methoxy derivative of R-4, exhibits a striking differential growth inhibitory effect in several different cancer cell lines. Noteworthy, MR-4 selectively eliminated cancer cells by inducing apoptosis and the apoptotic signalling may be triggered at mitochondria, which show very early perinuclear aggregation after MR-4 treatment. Taken together, our study suggests that resveratrol may serve as a useful chemical platform for generating more potent anticancer compounds targeting at mitochondria-mediated apoptotic pathway.

## MATERIALS AND METHODS

### Materials and chemicals

Dulbecco's modified Eagle's medium (DMEM) and fetal bovine serum (FBS) were obtained from Gibco BRL (Gaithersburg, MD, USA). [*α*-^33^P]dCTP was purchased from Perkin Elmer (Boston, MA, USA). Oligo-dT, poly-dA, dNTPs, Superscript™ II reverse transcriptase, Cot-1 DNA, MicroHyb hybridisation solution were purchased from Invitrogen Life Technologies (Baltimore, MD, USA). Anti-p53 antibody conjugated to horseradish peroxidase (polyclonal BMG-1B1) was purchased from Roche (Indianapolis, IN, USA), and antibodies for Bax, Bcl-2, and *β*-actin were from Santa Cruz iotechnology (Santa Cruz, CA, USA). Secondary anti-mouse (NIF 825) and anti-rabbit (NIF 824) conjugated to horseradish peroxidase were from Amersham Pharmacia (Piscataway, NJ, USA). Other chemicals were purchased from Sigma (St Louis, MO, USA).

### Preparation of resveratrol (R-3) and and 3,4,4′,5′-tetramethoxystilbene (MR-4).

Polyhydroxystilbenes and polymethoxystilbenes are designated as R or MR followed by a number indicating the number of hydroxyl or methoxyl groups (Lu *et al*, 2001). Whereas the natural occurring R-3 (3,5,4′-trihydroxy-*trans*-stilbene) contains only two hydroxyl groups in the first benzene ring, its analogues R-4 (3,4,5,4′-tetrahydroxy-*trans*-stilbene) and MR-4 (3,4,5,4′-tetramethoxy-*trans*-stilbene) contain, respectively, three hydroxyl and methoxyl groups. The second benzene ring is either hydroxylated (R-3 and R-4) or methoxylated (MR-4). The synthesis of resveratrol using 4-methoxybenzyl alcohol and 3,5-dimethoxy-benzaldehyde as the starting materials has been described (Bachelor *et al*, 1970; Drewes and Fletcher, 1974). Similar strategy was employed to prepare MR-4. The identity and purity of each of these compounds has been confirmed by thin layer chromatography, NMR and GC-mass spectroscopy.

### Cell culture and treatment

Different human normal cells including WI38 (normal lung fibroblasts), BJ-T (normal foreskin fibroblasts transfected with telomerase), and IMR-90 (normal fibroblasts) or human tumour cell lines including WI38VA (transformed lung fibroblasts), IMR-90SV (transformed fibroblast), HeLa (cervix cancer), LNCaP (prostate cancer), HT-29 (colon cancer), and HepG2 (hepatoma cells) were obtained from the National Institute on Aging Repository (Coriell Institute for Medical Research, Camden, NJ, USA). Cells were cultured in Dulbecco's modified Eagles medium (DMEM) supplemented with 10% FBS at 37°C in a humidified, 10% CO_2_ atmosphere. Cells were subcultured in culture flasks (Falcon, Becton-Dickinson, Franklin Lakes, NJ, USA) and passaged every 3 days. Before experiments, cells were seeded either in 88, 35-mm culture dishes or in 24-well plates (Falcon, Becton-Dickinson, Franklin Lakes, NJ, USA) as indicated for the different assays to establish a confluent monolayer for untreated control cells at the end of the experiment. Different stock solutions of R-3 or MR-4 in DMSO were applied to the medium.

### Cellular proliferation assays and morphological analysis

Cell proliferation was measured by the MTT (3(4,5-dimethylthiazol-2-yl)-2,5-diphenyl-tetrazolium-bromide) method, crystal violet staining, or by cell counting. For the MTT- and crystal violet assay, cells were treated in 24-well plates for 5 days, whereas for the counting cell numbers were determined at designated time points. The MTT-assay, based on the conversion of the tetrazolium salt MTT to blue formazan by mitochondrial dehydrogenase, measures mitochondrial damage (Berg *et al*, 1990). Owingto the concern that MTT assay may yield false-positive results for certain cell types when treated with flavonoids or polyphenols (Bernhard *et al*, 2003), we also included the crystal violet dye staining assay in this study (Lu *et al*, 2001). Colour development was documented by a scanner (UMAX, Astra 2200). For the counting method cells were analysed by phase contrast light microscopy and documented using a digital camera system (MDS 120, Kodak, Emeryville, CA, USA). For morphological analysis, cells were treated in 35-mm cell culture dishes for 5 days. Morphological analysis was performed by phase contrast light microscopy and documented by the digital camera system (MDS 120, Kodak, Emeryville, CA, USA).

### DNA fragmentation assay

After treatment in 60-mm culture dishes, cells were harvested and resuspended in 500 *μ*l of lysis buffer (0.5% (w v^−1^) sodium lauryl sarkosinate+10 mM EDTA+0.5 mg ml^−1^ proteinase K+0.1 mg ml^−1^ RNase A in 50 mM Tris-Base, pH 8.0). After an incubation for 40 min at 37°C, proteins were precipitated in 1 M NaCl at 4°C for 1 h, followed by a centrifugation (10 000 × *g* for 10 min). DNA from the supernatant was then extracted with a phenol/chloroform/isopropanol mixture (25 : 24 : 1, pH 8.0), precipitated by ethanol (70%), and dried in a Speed Vac. For separation, DNA pellets were resuspended in water (20 *μ*l) and 10 *μ*l of DNA laddering loading buffer (1% low-gelling temperature agarose (w v^−1^)+0.25% bromphenol blue (w v^−1^)+40% sucrose in 10 ml EDTA, pH 8.0), which was heated to 100°C, previously. After loading onto a 2% agarose gel, the samples were solidified for 1 h. Thereafter, DNA samples were separated by electrophoresis, stained by ethidium bromide and visualised under UV illumination.

### Caspase activation assay

The effect of MR-4 on caspase activation was analysed by CaspACE™FITC-VAD-FMK *in situ* marker (Promega, Madison, WI, USA), which is a fluoroisothiocyanate (FITC) conjugate of the cell permeable caspase inhibitor VAD-FMK. The structure allows delivery of the inhibitor into the cells where it binds to activated caspases. Cells were seeded on coverslips 2 days before experiment. After treatment, cells on coverslips were transferred onto a 35-mm dish containing 1 ml of medium. Then, 2 *μ*l of CaspACE-solution were applied to the medium to achieve a final concentration of 10 *μ*M. After an incubation for 20 min, cells were analysed by fluorescent microscopy using an excitation wavelength of 480 nm.

### Reverse transcription–polymerase chain reaction (RT–PCR)

After treatment, cells were harvested at indicated times and the total RNA was prepared using RNeasy™ Total RNA Kit (Qiagen, Chatsworth, CA, USA). Then, total RNA (1 *μ*g) was reverse transcribed into cDNA by incubating with SuperScriptTM RNase H reverse transcriptase (Invitrogen Life Technologies, Baltimore, MD, USA) using oligo(dT)_12−18_ as primer (Invitrogen Life Technologies, Baltimore, MD, USA). For PCR amplification, gene specific primers, both sense and antisense, were used. The sequences of these primers were:

(Bax sense)=5′-CTg ACA TgT TTT CTg ACg gC-3′;

(Bax antisense)=5′-TCA gCC CAT CTT CTT CCA gA-3′;

(Bcl-2 sense)=5′-ACT TgT ggC CCA gAT Agg CAC CCA g-3′;

(Bcl-2 antisense)=5′-gCg ACT TCg CCg AgA TgT CCA gC-3′;

(GAPDH sense)=5′-TgA AgC TCg gAg TCA Acg gAT TTg-3′;

(GAPDH antisense)=5′-CAT gTg ggC CAT gAg gTC CAC CAC-3′.

PCR conditions were chosen to ensure that the yield of the amplified product was linear with respect to the amount of input RNA. The expression of glyceraldehyde-3-phosphate dehydrogenase (GAPDH), a housekeeping gene, was used as an internal control. The PCR products were analysed by electrophoresis on a 1% agarose gel and visualised by ethidium bromide staining.

### SDS–PAGE and Western blot

After treatment, cells were harvested, washed, heated, and sonicated in lysis buffer (5 mM Tris-base (pH 6.8), 5% 2-mercaptoethanol, 3% sodiumdodecylsulfate (SDS), 10% glycerol). After centrifugation (18000 × *g* for 10 min at 20°C), equal amounts of protein were separated by SDS–PAGE (10%) and transferred onto a nitrocellulose membrane for Western blot analysis. Blots were blocked with 1% skim milk and 0.2% Tween 20 in phosphate-buffered saline (PBST) for 0.5 h at room temperature. Primary antibodies used were: anti-p53 antibody conjugated to horseradish peroxidase (polyclonal BMG-1B1, Roche, Indianapolis, IN, USA), anti-Bax (rabbit polyclonal, sc-526), anti-Bcl-2 (mouse monoclonal, sc-509), and anti-*β*-actin (mouse monoclonal, sc-8432), all from Santa Cruz Biotechnology, Santa Cruz, CA, USA. Secondary anti-mouse (NIF 825) and anti-rabbit (NIF 824) conjugated to horseradish peroxidase were from Amersham Pharmacia (Piscataway, NJ, USA). Primary and secondary antibodies were incubated for 1 h followed by three washes in PBST. Immuno-complexes were detected with the ECL Plus Western blot detection kit (Amersham Pharmacia, Piscataway, NJ, USA).

### Rhodamine 123 fluorescence assay

Morphology of mitochondria was analysed using the cationic fluorophore rhodamine 123 which specifically localises mitochondria in living cells. Due to the negative electric potential across the mitochondrial membrane rhodamine 123 transfers electrophoretically into the mitochondria (Johnson *et al*, 1980). Before experiments, cells were grown on glass coverslips in 35-mm culture dishes for 2 days. After treatment, 4 *μ*l of rhodamine 123 stock solution (1.5 mM in medium containing 10% DMSO) were added to the dishes to achieve a final concentration of 1.5 *μ*M and incubated for 20 min. Then, coverslips were washed twice with medium to remove extracellular rhodamine. Cells were analysed either by phase contrast microscopy to monitor cell morphology or by fluorescence microscopy to determine mitochondrial morphology. For fluorescence microscopy, excitation and emission wavelength were 480 or 525 nm, respectively.

## RESULTS

### The effect of MR-4 and R-3 on normal and transformed human fibroblasts

**Figure 1:**
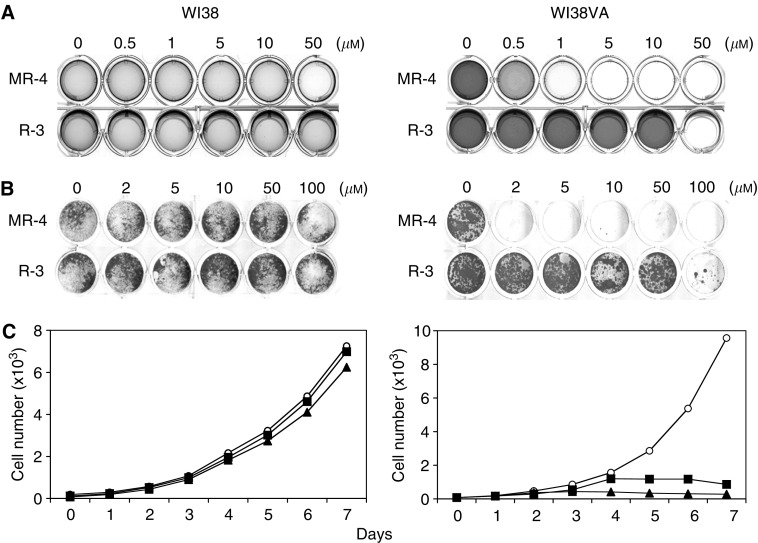
Growth inhibition induced by MR-4 and R-3 in normal and transformed fibroblasts. Normal (WI38; left panel) or transformed fibroblasts (WI38VA; right panel) were treated by different concentrations of MR-4. Then, cell growth was either analysed by (**A**) the MTT-assay and (**B**) the crystal violet assay after 5 days or by (**C**) cell counting after different time points as described in Material and Methods. For cell counting, cells were treated by 0.5 *μ*M (closed squares), 1 *μ*M (closed triangles), or were left untreated as controls (open circles) and counted at indicated times. Results obtained by the MTT- or crystal violet assay were documented by a scanner (**A**, **B**) or expressed as cell numbers for the cell counting method (**C**). Representative data of three independent experiments are shown.

We first compared the effects of MR-4 and R-3 on the growth of normal and transformed human fibroblasts using the MTT-reduction assay (Figure 1A). We also employed the crystal violet staining assay to monitor cell proliferation (Figure 1B). We found that both methods yielded qualitatively similar results. Human fibroblast WI38 and the virally transformed WI38 (termed WI38VA) were used as a model for paired normal and cancer cells in this study. Both cell lines exhibit comparable growth rate with the doubling time about 24 h. For WI38 cells, both MR-4 and R-3 showed little or no inhibitory effect up to 50–100 *μ*M as determined by the MTT- or crystal violet assay. For WI38VA cells, MR-4 completely inhibited the growth at 1–2 *μ*M, whereas R-3 was much less potent, being effective only at concentrations greater than 50 *μ*M. Thus, MR-4 was about 100-fold more potent than R-3 in inhibiting the growth of transformed WI38VA cells. In addition to the MTT- and crystal violet assay, we also quantified the effect of MR-4 on growth rates of WI38 and WI38VA cells by viable cell counting (Figure 1C). When exposed to 0.5 or 1 *μ*M, the differential growth inhibitory effect was evident 3 days after treatment. In summary, all three proliferation assays demonstrate that MR-4 exhibited a clear differential growth inhibitory effect toward transformed human cells. Moreover, MR-4 was about 50- to 100-fold more potent than R-3 in inhibiting the growth of transformed cells.

### Effects of MR-4 on other normal and cancerous human cells

**Figure 2:**
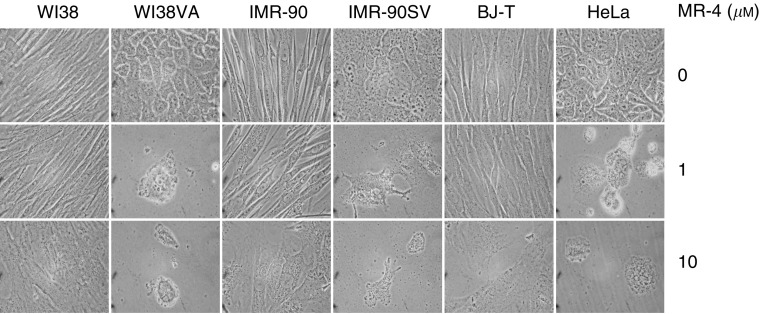
Morphology of various normal and cancer cells after treatment by MR-4. Different normal cell strains including WI38 (normal lung fibroblasts), BJ-T (normal foreskin fibroblasts transfected with telomerase), and IMR-90 (normal fibroblasts) as well as transformed or cancer cell lines including WI38VA (SV40 virally transformed lung fibroblasts), IMR-90SV (SV40 virally transformed fibroblast), and HeLa (cervix cancer) were treated with 1 or 10 *μ*M of MR-4 for 5 days. Morphological analysis was performed by phase contrast light microscopy.

The differential growth inhibitory effect of MR-4 on transformed WI38VA cells prompted us to examine whether this finding also holds for other cancer cell types. Figure 2 shows the phase contrast micrographs of three normal, two virally transformed (WI38VA, IMR-90SV), and one cancer cell line (HeLa) in the presence of 1 or 10 *μ*M of MR-4. At both concentrations MR-4 inhibited the growth of transformed and cancer cells. MR-4 also inhibited the growth of LNCaP prostate cancer cells, HT-29 colon cancer cells, and HepG2 hepatoma cells with the IC_50_ values in the range of 1–5 *μ*M (data not shown). On the other hand, MR-4 did not significantly suppress the growth of normal cells such as WI38, IMR90, and BJ-T. Although MR-4 did not affect the growth and viability of the normal cells, it did cause slight changes in cell morphology at high concentrations (>10 *μ*M). These results suggest that the differential growth inhibitory effect of MR-4 appeared to be general for transformed and cancerous cells.

### Effect of MR-4 on apoptosis in normal and transformed human fibroblasts

**Figure 3:**
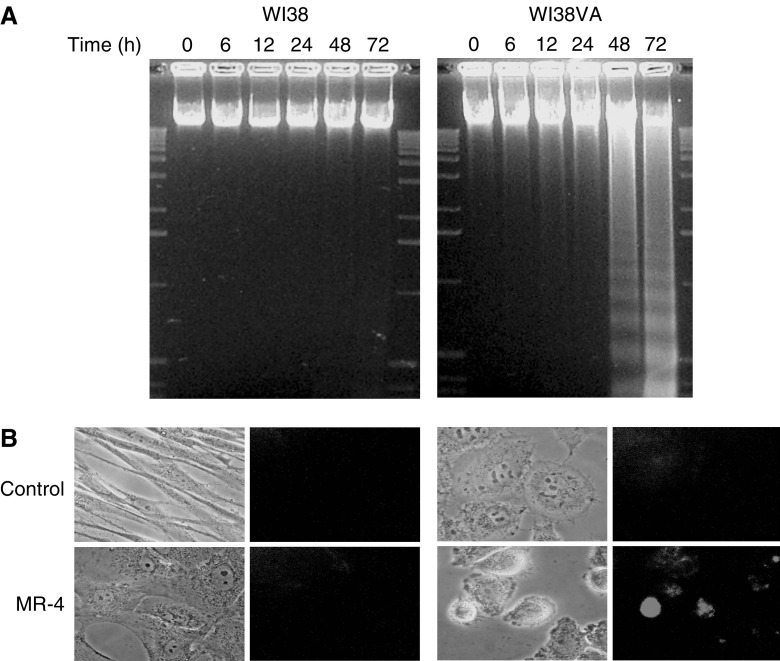
Analysis of apoptotic cell death by (**A**) DNA fragmentation and (**B**) caspase activation assay in normal and transformed fibroblasts. Confluent WI38 or WI38VA cultures were treated with MR-4 (50 *μ*M) and processed for apoptosis assay. (**A**) DNA laddering. DNA was isolated from cells at indicated time points and analysed by agarose electrophoresis and ethidium bromide staining. (**B**) Caspase activation-assay. After 48 h of MR-4 treatment, the effect on caspase activation was analysed by the CaspACE™ *in situ* marker FITC-VAD-FMK, which specifically binds to the activated caspases. After an incubation of 20 min with the fluorophore, cells were examined by fluorescent microscopy. For each treatment, representative phase contrast (left) and fluorescence (right) micrographs were taken. Upper and lower panels represent controls or MR-4 treated cells, respectively.

Abundant literature evidence suggests that the antiproliferative effect of resveratrol is due to its ability to induce apoptosis (Gusman *et al*, 2001). It is therefore likely that the differential growth inhibitory effect of MR-4 may be due to selective induction of apoptosis in transformed cells, but not in normal cells. To examine this possibility, we first determined whether MR-4 could elicit DNA laddering, a signature event of apoptotic process (Compton, 1992; Green and Reed, 1998; vanGurp *et al*, 2003). Figure 3A shows an extensive DNA fragmentation in transformed WI38VA cells 48 h after MR-4 treatment. In contrast, DNA laddering was not observed in normal WI38 cells throughout the entire time course. Caspases are a family of aspartyl-specific cysteine proteases that play a key role in transmitting and executing a whole array of apoptotic signals (Budihardjo *et al*, 1999). We therefore also examined whether MR-4 had differential effects on global caspase activation. Figure 3B shows that the activation of caspases occurred only in WI38VA cells following MR-4 treatment, but not in WI38 cells. These results indicate that MR-4 selectively induced apoptotic signalling pathways in transformed cells but not in normal cells.

### Effect of MR-4 on the expression of apoptosis-related genes in normal and transformed human fibroblasts

Mitochondria-mediated apoptotic pathways involve modulation of the expression of genes such as p53, the key upstream player in apoptosis, and Bcl-2 family genes (Green and Reed, 1998; Sheikh and Fornace, 2000). The Bax/Bcl-2 ratio has been considered to be a rheostat of the apoptosis because it is related to the control of the permeability of mitochondrial membranes via oligomerisation mechanism (Korsmeyer *et al*, 1993; Korsmeyer, 1999). Induction of p53, both at mRNA and protein level, in cells treated with resveratrol is thought to be the major cause for apoptosis (Huang *et al*, 1999). To examine whether MR-4 affects p53 and Bcl-2 family genes, we performed RT–PCR and Western blot analysis in normal and transformed fibroblasts.

**Figure 4:**
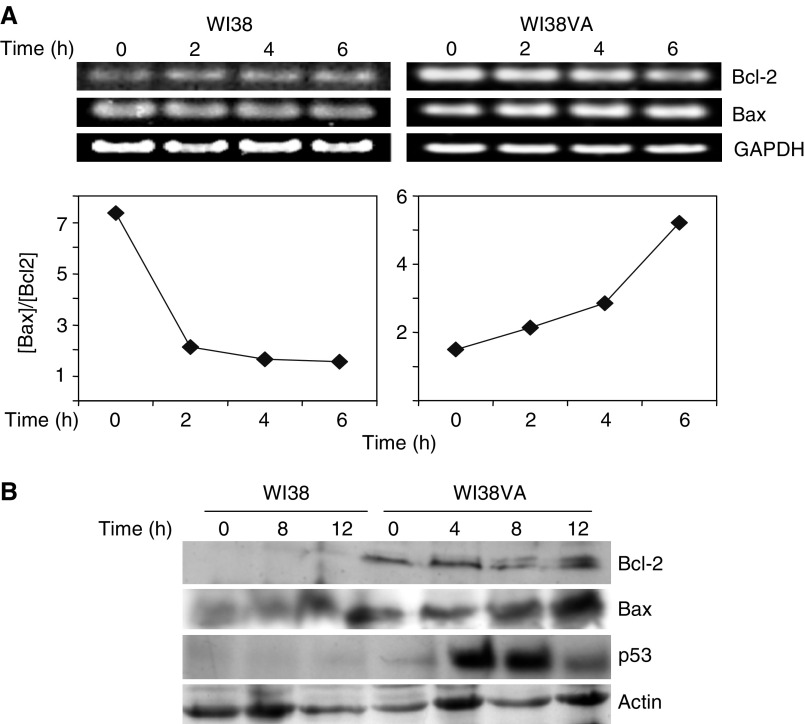
Effect of MR-4 on the expression of apoptotic genes in normal and transformed fibroblasts. (**A**) RT–PCR analysis. MR-4 (50 *μ*M) was applied for 0, 2, 4, or 6 h to WI38 or WI38VA. The induction of Bax and Bcl-2 was analysed by RT–PCR using 1 *μ*g of total RNA. Glyceraldehyde-3-phosphate dehydrogenase (GAPDH) was used as internal standard. Bax/Bcl-2 ratio was determined by densitometry and normalised to GAPDH. (**B**) Western blot analysis. MR-4 (50 *μ*M) was applied for 0, 4, 8, or 12 h. Cells were harvested at indicated times and equal amount of cellular protein (30 *μ*g) was analysed by SDS–PAGE. Western blot analysis was performed using specific antibodies against Bax, Bcl-2, p53, and actin as described in Materials and Methods.

Figure 4A shows that at the mRNA level, the ratio of Bax and Bcl-2 gene expression increased in WI38VA cells and the value reached to four-fold 6 h after the MR-4 treatment while the Bax/Bcl-2 ratio in WI38 cells decreased after MR-4 treatment. When analysed at protein level, we observed a prominent increase of p53 by more than 10-fold in WI38VA cells within 4 h (Figure 4B). Moreover, we noticed that although the Bcl-2 level in WI38VA cells treated with MR-4 did not show significant change, an additional protein band appeared, most likely due to phosphorylation of Bcl-2 (Haldar *et al*, 1995). Consistent with the increase in the Bax/Bcl-2 mRNA ratio, Bax protein level also increased in WI38VA cells 8–12 h after MR-4 treatment (Figure 4B). In contrast, the p53, Bcl-2 and Bax level in WI38 cells were barely or much less detectable even after 12 h of MR-4 treatment (Figure 4B). These results show that MR-4 selectively induced an increase in p53 and Bax/Bcl-2 ratio, consistent with the notion that MR-4 treatment activated mitochondria-mediated apoptotic pathways only in transformed cells.

### Mitochondrial morphology and distribution in normal and transformed human fibroblasts

**Figure 5:**
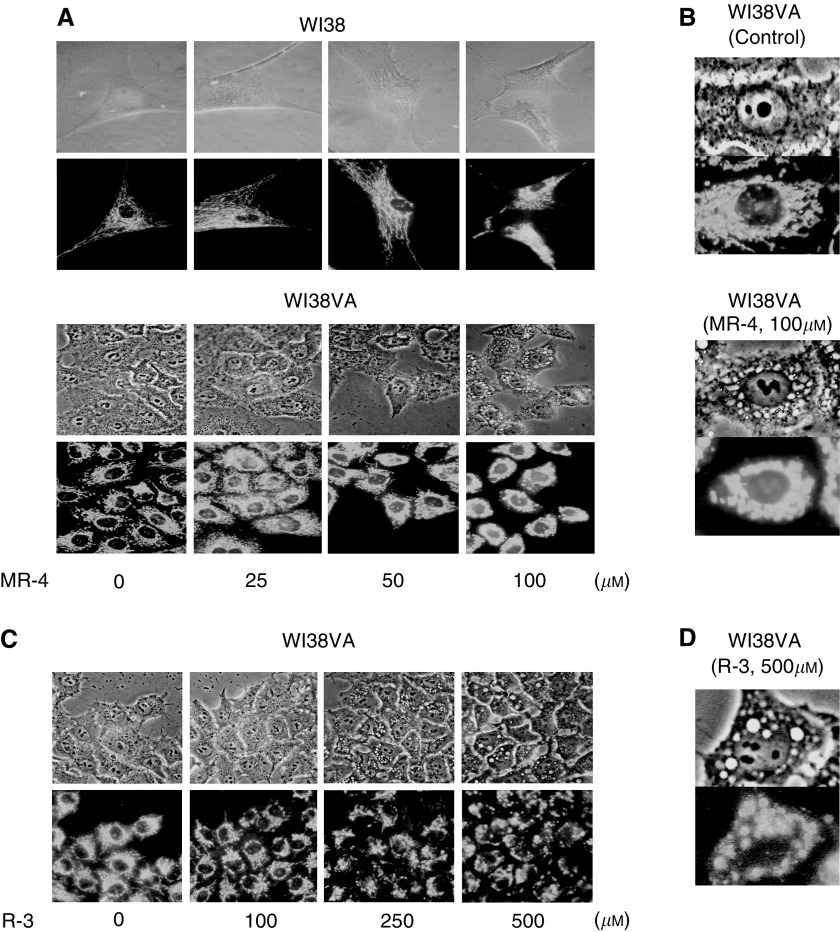
Effect of MR-4 and R-3 on the mitochondrial morphology in normal and transformed fibroblasts. (**A**) Dose–response study of the effect of MR-4 on mitochondrial localisation in WI38 and WI38VA cells. Cells were treated with different concentrations of MR-4 for 3 h and then stained with rhodamine 123 for 20 min. Cells were then analysed either by phase contrast (top of each panel) or fluorescence microscopy (bottom of each panel) at a final magnification of × 1000. (**B**) Effect of MR-4 on WI38VA cells. Cells treated without (control) or with MR-4 (100 *μ*M) for 3 h and analysed at a magnification of × 5000. (**C**) Dose–response study of the effect of R-3 on mitochondrial localisation in WI38VA cells. Cells were treated with R-3 at indicated concentrations for 3 h and then stained with rhodamine123 for 20 min and then analysed by phase contrast (top panels) and fluorescent (bottom panels) microscopic examination. (**D**) Effect of R-3 on WI38VA cells. Cells were treated with R-3 at 500 *μ*M for 3 h and then examined under phase contrast (upper) and fluorescent (bottom) microscopy at a magnification of × 5000.

The upregulation of the Bax/Bcl-2 ratio and p53 prompted us to analyse possible effect of MR-4 on the mitochondrial morphology and distribution. Cationic fluorophore rhodamine 123 stains intact mitochondria rather specifically (Johnson *et al*, 1980). We therefore employed rhodamine 123 to monitor possible changes in mitochondrial localisation and clustering in normal and transformed fibroblasts following the MR-4 treatment. We found a drastic change in mitochondrial morphology only in WI38VA cells treated by MR-4. As shown in Figure 5A, MR-4 induced a dose-dependent perinuclear clustering of mitochondria in transformed WI38VA cells, but not in their normal counterpart, WI38 cells. Figure 5B shows the effect of MR-4 on mitochondrial distribution in WI38VA cells at higher magnification. Mitochondria in control WI38VA cells were distributed throughout the cell body, but became aggregated and clustered around the nucleus (perinuclear clustering) after only 3 h after MR-4 treatment (Figure 5B, bottom *vs* top). In contrast, the web-like cellular distribution of mitochondria in normal cells was not significantly affected by MR-4 up to 100 *μ*M. In a time course study using only 5 *μ*M MR-4, we observed prominent aggregation of mitochondria in WI38VA cells 6 h after the treatment (data not shown). When the effect of R-3 was analysed (Figure 5C), we found that R-3 did cause apoptosis and clustering of mitochondria in WI38VA cells, but only at much higher concentrations, up to 500 *μ*M. Figure 5D shows the mitochondrial clustering induced by R-3 at 500 *μ*M in WI38VA cells. The clustering induced by R-3 was not as prominent and clear as that observed with MR-4 (Figure 5D *vs* Figure 5B). The effect of MR-4 on mitochondrial clustering was quite dramatic and it was evident even without fluorescent staining (Figure 5B). The observation that MR-4 significantly altered the morphology and distribution of mitochondria in transformed fibroblasts suggests that MR-4 may specifically evoke death signals originating from mitochondria in transformed cells. The fact that R-3 caused apoptosis and mitochondrial clustering only at much higher concentration suggests that MR-4 and R-3 may share a common target site, but the action of MR-4 is more specific and potent.

## DISCUSSION

It has been shown that resveratrol attenuates carcinogenesis or tumour progression both *in vitro* and *in vivo*, and is likely the key ingredient for the health beneficial effect of grape wine (Jeandet *et al*, 1991; Jang *et al*, 1997; Elattar and Virji, 1999; Hsieh and Wu, 1999; Lu and Serrero, 1999; Surh *et al*, 1999; Tsan *et al*, 2000; Gusman *et al*, 2001; Ding and Adrian, 2002; Narayanan *et al*, 2003; Zhou *et al*, 2003). Although the antiproliferative effect of resveratrol seems to be due to the induction of apoptosis (Hsieh and Wu, 1999; Surh *et al*, 1999; Tsan *et al*, 2000; Ding and Adrian, 2002; Zhou *et al*, 2003), current research on the use of resveratrol as potential cancer chemoprevention agent has been largely limited to the study with only cancer cells. In an attempt to determine whether chemical modification of the stilbene backbone of resveratrol may further enhance its biological activity and differentiate cancer cells from their normal counterparts, we have synthesised and tested a number of hydroxy- and methoxy-stilbenes (Lu *et al*, 2001). Among them, MR-4 is by far not only the most potent one but also is the one that exhibits the most striking differential growth inhibitory effect against cancer cells (Figures 1 and 2). As previously noted (Chen *et al*, 1998; Lu *et al*, 2001; Gosslau and Chen, 2004), when tested as a potential anticancer agent it is desirable that the compound has minimal detrimental effect on the growth or survival of normal cells. In this regard, MR-4 may be superior to natural resveratrol as a candidate for chemoprevention agent. Nevertheless, further work on toxicity is needed to establish this possibility. Since MR-4 did not exhibit any antioxidant activity as demonstrated by the thiobarbituric acid assay (A Gosslau and KY Chen, unpublished result), the enhanced anticancer activity of MR-4 does not appear to be related to antioxidation, as known to be associated with various polyphenol compounds. The fact that R-4 and MR-4, but not R-3 or MR-3 (the trimethoxy derivative of R-3), exhibits differential growth inhibitory effect (Lu *et al*, 2001 and Figures 1 and 2) suggests that structure-related mechanisms (an additional hydroxyl or methoxyl group) may be crucial in revealing the target sites that differentiate cancer cells from their normal counterparts. On the other hand, we cannot rule out the possibility that the differences between the effects of R-3 and MR-4 reflect differences in the uptake of these compounds.

The close correlation between the growth inhibition (Figures 1 and 2) and the effect of MR-4 on apoptosis in transformed cells, as determined by morphological damage, DNA laddering and caspase activation (Figure 3), suggest that the target sites of MR-4 could be any components involved in apoptotic pathway or the upstream events that lead to apoptosis. The tumour suppressor protein p53 is known to function as a gatekeeper controlling critical cell cycle checkpoints (Levine, 1997) and also act as a key player in mediating apoptosis through modulating the Bax to Bcl-2 ratio and the regulation of mitochondrial ROS generation (Miyashita and Reed, 1995; Johnson *et al*, 1996; Levine, 1997; Yin *et al*, 1997; Sheikh and Fornace, 2000; Pietenpol and Stewart, 2002). The findings that MR-4 led to the induction of p53, increases in Bax and Bax/Bcl-2 ratio (Figure 4) and caused a rapid perinuclear clustering of mitochondria (Figure 5) in transformed cells, but not in normal cells, could explain the selective apoptotic effect of MR-4 in cancer cells. The appearance of Bcl-2 doublet in MR-4 treated WI38VA cells (Figure 4B) also suggests that the phosphorylation of Bcl-2 may contribute to the apoptotic process in transformed cells, as it has been shown that Bcl-2 phosphorylation not only inactivates itself but also facilitates permeabilisation of mitochondria membrane (Haldar *et al*, 1995).

In accordance with the notion that MR-4 may trigger the mitochondria-mediated apoptosis in transformed cells, we observed prominent condensation and clustering of mitochondria around the nucleus in cancer cells, but not in normal cells after MR-4 treatment (Figure 5A and B). This dramatic morphological change becomes obvious within 3 h after treatment and represents one of the earlier events induced by MR-4. Our observation that R-3 also induced clustering of mitochondria, although at much higher concentrations as compared to MR-4 (Figure 5C and D), suggests that MR-4 and R-3 may share a common target site. Other investigators have reported that mitochondria-mediated apoptosis sometimes is accompanied with morphological changes and redistribution of mitochondria in apoptotic cells (Desagher and Martinou, 2000). The mechanism of clustering is unknown. The possible causal relationship between mitochondria clustering and apoptosis needs to be clarified. One possible scenario is that MR-4 causes mitochondrial clustering and depolarisation in cancer cells, which results in the release of proapoptotic factors and triggers apoptosis. We observed the formation of crystal-like structures specifically around mitochondria of transformed fibroblasts treated by MR-4 (Figure 5B). The identification of the nature of these structures may help to resolve the apoptotic mechanisms induced by MR-4 in cancer cells. In addition, other targets of MR-4 particularly can be further investigated by microarray technology.

Taken together, our data show that the resveratrol analogue MR-4 exhibits potent and selective growth inhibitory effect to cancer cells. The specific induction of perinuclear clustering of mitochondria and the activation of the mitochondria-mediated apoptotic pathway seem to be the major cause for the striking differential inhibitory effect of MR-4. Identification of the target of MR-4 in cancer cells will be the key for our understanding of the action of MR-4. A major challenge in cancer therapy is to search or synthesise drugs that kill tumour cells while preserve normal cells. In this regard, polyhydroxystibene may serve as a useful chemical platform for developing more potent and selective anticancer reagents.
